# Navigating challenges and workarounds: A qualitative study of healthcare and support workers' perceptions on providing care to people seeking sanctuary

**DOI:** 10.1111/hex.14061

**Published:** 2024-04-28

**Authors:** Ashrafunnesa Khanom, Bridie A. Evans, Wdad Alanazy, Lauren Couzens, Lucy Fagan, Rebecca Fogarty, Ann John, Talha Khan, Mark R. Kingston, Samuel Moyo, Alison Porter, Gillian Richardson, Grace Rungua, Victoria Williams, Helen Snooks

**Affiliations:** ^1^ Swansea University Medical School, ILS 2 Swansea University Swansea UK; ^2^ Public Health Wales Cardifff UK; ^3^ University College Cork Cork Ireland; ^4^ British Red Cross London UK; ^5^ Public Contributor Swansea UK; ^6^ Displaced People in Action (DPIA) Cardiff UK

**Keywords:** asylum seekers, delivery of healthcare, health personnel, primary healthcare, qualitative research refugees

## Abstract

**Background:**

Healthcare and support workers play a pivotal role in delivering quality services and support to people seeking sanctuary who have experienced poor physical and mental health linked to previous trauma, relocation and loss of freedoms. However, they often encounter various challenges in their daily work, ranging from communication barriers to resource constraints. This qualitative study seeks to delve into the perspectives of healthcare and support workers' experience of workarounds, employed to overcome barriers to providing care.

**Aim:**

This study aims to describe healthcare providers', practitioners' and health and third sector support workers' views on barriers and workarounds to providing care for people seeking sanctuary, to inform policy and practice.

**Design:**

A qualitative study was carried out using semi‐structured telephone interviews.

**Setting:**

This study focused on primary, secondary, community and specialist National Health Service (NHS) support services for people seeking sanctuary in Wales, United Kingdom (2018).

**Method:**

We interviewed 32 healthcare providers, practitioners and support workers employed by primary care and third sector organisations. Our approach involved obtaining verbal informed consent before digitally recording and transcribing all interviews. To analyse the data, we used the Four Levels of Change for Improving Quality model as a guiding framework for interpretation.

**Results:**

Our study findings reveal that certain respondents expressed challenges in meeting the needs of people seeking sanctuary; notably, their experience of delivering care differed by care settings. Specifically, those involved in providing specialist NHS care believed that there was room for improvement. Mainstream primary, secondary and community health practitioners faced limitations due to resource constraints and lacked tailored information to address the unique circumstances and needs of sanctuary seekers. To address these gaps, workarounds emerged at both individual and local levels (team/departmental and organisational level). These included establishing informal communication channels between providers, fostering cross service collaboration to fill gaps and adapting existing services to enhance accessibility.

**Conclusion:**

Understanding healthcare providers', practitioners' and support workers' perspectives offers invaluable insights into ways to enhance healthcare delivery to sanctuary seekers. Acknowledging challenges and harnessing innovative workarounds can foster a more effective and compassionate service for this vulnerable population.

**Patient or Public Contribution:**

The HEAR study actively involved public contributors in the design, delivery and dissemination of the research. Two public contributors (S. M. and G. R.) who had personal experience of seeking asylum served as study co‐applicants. They played pivotal roles in shaping the research by participating in its development and securing funding. Alongside other co‐applicants, S. M. and G. R. formed the Research Management Group, overseeing study delivery. Their contributions extended to strategic decision‐making and specific feedback at critical junctures, including participant recruitment, data collection, analysis and reporting. Additionally, S. M. and G. R. were instrumental in recruiting and supporting a team of peer researchers, enhancing respondent participation among people seeking sanctuary. To facilitate effective public involvement, we provided named contacts for support (A. K. and R. F.), research training, honoraria, reimbursement of expenses and accessible information in line with best practice.

## 1 INTRODUCTION

International conflicts and human rights abuses have contributed to the growing number of people seeking sanctuary throughout the world.^1^ Those people who seek sanctuary in the United Kingdom are referred to as ‘asylum seekers’.^2^ In 2022, over 4.9 million individuals sought asylum globally. Another 32.5 million people attained refugee status, with nearly half of them being under the age of 18 years.^1^

Persecution and the arduous journey experienced by people seeking sanctuary can impact their health. They often present with mental health problems, infections, chronic diseases, trauma from past experiences and stress related to settling into a new country.^3^, ^4^, ^5^ To address their healthcare needs, the United Nations High Commissioner for Refugees (UNHCR) Executive Committee has set minimum standards for host nations. These standards aim to ensure basic healthcare access for all people seeking sanctuary,^6^, ^7^ thereby preventing the spread of infectious disease, mitigating health deterioration^8^ and alleviating the burden on secondary care systems.^9^, ^10^

In the United Kingdom, people seeking sanctuary struggle to communicate in English, navigate unfamiliar health systems and experience discrimination.^11^ Asylum seekers face additional issues such as state surveillance, along with movement and employment restrictions until their asylum claim is decided by the Home Office.^11^, ^12^, ^13^, ^14^ Most are required to report to a Home Office building or a local police station on a regular basis and failure to do so could impact their asylum claim. Once asylum is granted, asylum seekers transition to refugee status and are free to choose their place of residence, seek employment and access public funds. While primary and secondary healthcare is free at the point of use for those seeking sanctuary in the United Kingdom, pathways to care differ based on immigration law, generating uncertainty among health professionals on the criteria for determining eligibility to receive care.^15^ In Scotland and Wales, both asylum seekers and refused asylum seekers are entitled to free primary, emergency and secondary healthcare. In England, refused asylum seekers are only entitled to free secondary healthcare if they receive support from the Home Office, local authority or fall under Part 1 of the Care Act 2014.^16^ However, refused asylum seekers can receive free NHS hospital treatment for accident and emergency care and the diagnosis and treatment of infectious diseases.

Considering the broader context of asylum and resettlement policy and process, those who provide healthcare to people seeking sanctuary encounter several challenges. These include communication barriers and cultural differences, which can hinder effective healthcare interactions.^10^ Resource constraints in terms of access to healthcare facilities and timely access to healthcare providers can impact the availability and quality of care. Finally, legal restrictions such as reporting or relocation policies can create barriers to accessing optimal care.^2^, ^3^, ^10^, ^15^

Previous research has primarily focused on the perspectives of care providers within primary care environments.^11^, ^12^, ^13^ However, our study takes a broader approach. We report on insights from a range of healthcare providers, practitioners and support workers including those from primary care and third sector. By doing so, we aim to identify barriers and enablers to providing care for people seeking sanctuary and to develop strategies to improve access, thereby informing policy and practice decisions. This paper forms part of a broader mixed‐methods study commissioned by Public Health Wales^17^, ^18^ in response to the Welsh Government's people seeking sanctuary report, ‘I used to be someone’^19^ to aid NHS compliance to the Wellbeing of Future Generations (Wales) Act 2015,^20^ for a more equal and healthier Wales.

## 2 METHOD

### 2.1 Conceptual framework

**Figure 1:**
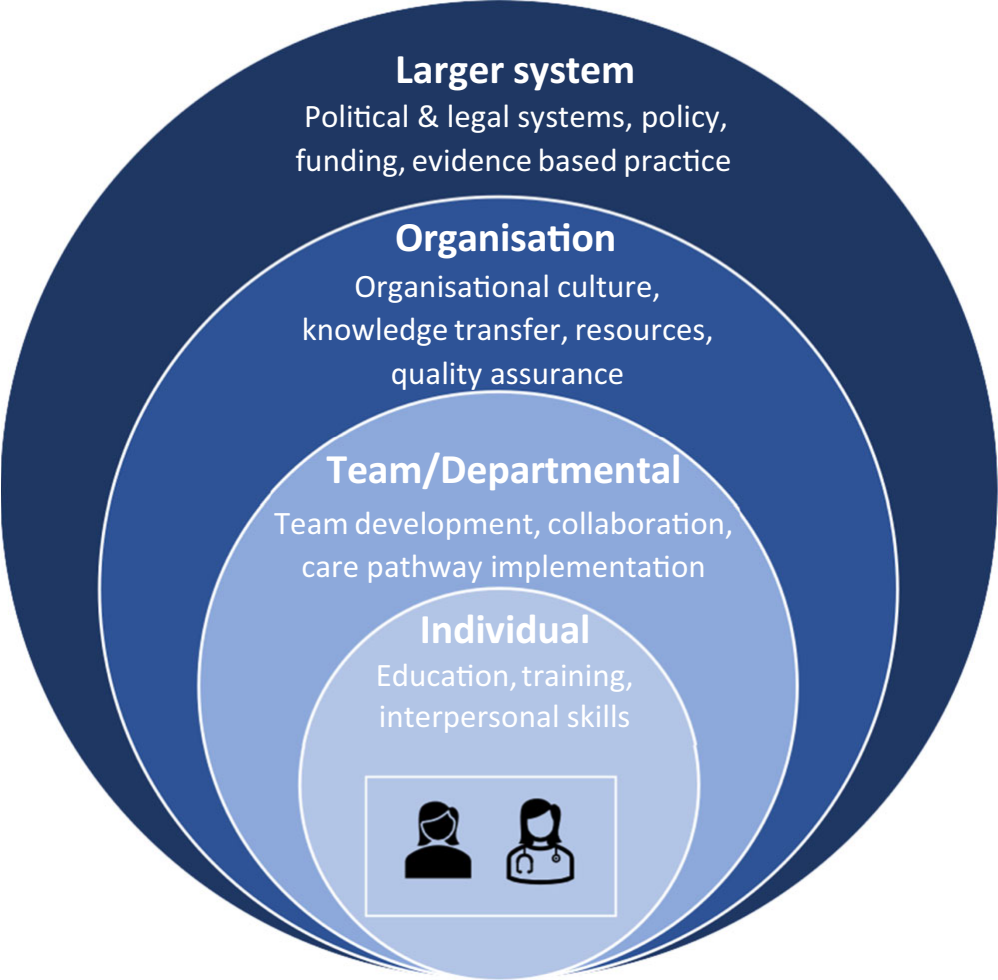
Ferlie and Shortell,^23^ Four Levels of Change for Improving Quality.

In a previous paper related to this study,^21^ we outlined the barriers to accessing care as reported by people seeking sanctuary using a conceptual model developed by Levesque et al.^22^ In this paper, we endeavour to report on the challenges faced by healthcare providers in delivering care to people seeking sanctuary and to identify key levers that could drive positive change. We used the Four Levels of Change for Improving Quality model^23^ as a framework to interpret our qualitative interview data. The model can be applied to a healthcare context, where innovation could be mobilised across the system at various levels to facilitate effective change (see Figure 1).

#### 2.1.1 Individual Level 1

Where individual healthcare or support workers adopt and implement change by reviewing their behaviours and practices.

#### 2.1.2 Team/departmental Level 2

Where healthcare teams or departments focus on collaboration, communication and coordination to integrate change across their specific areas of responsibility.

#### 2.1.3 Organisational Level 3

Where the entire organisation embraces change that permeates at every level of the organisation. It aligns its mission and values with these changes and actively supports their implementation for meaningful impact and sustainable progress.

#### 2.1.4 Systems Level 4

Where socioeconomic, legal and political actors align their efforts to ensure that broader systemic changes are consistent and effective across sectors.

While change is possible at any level, Ferlie and Shortell^23^ believe that transformative change and positive sustainable outcomes are achieved when different levels within a system or organisation work collaboratively to enact change across the entire system.

## 3 SETTING

People seeking sanctuary have been dispersed to Wales since 2001 and comprise around 0.65% of the Welsh population. All 22 local authority areas house people seeking sanctuary including those settled through the Syrian Vulnerable Persons Resettlement Scheme and resettlement schemes for Afghani and Ukrainian sanctuary seekers.^24^ Processes for receiving and providing healthcare to refugees differ from those for asylum seekers arriving in Wales (see Box 1). Just over 10,000 refugees are thought to live in Wales^19^, with an additional 4192 Ukrainian refugees resettled since 2022.^24^ Approximately 5617 asylum seekers were provided ‘Section 5’ (subsistence only) and ‘Section 95’ (accommodation and subsistence) support by the Home Office between 1 April 2021 and 31 March 2022.^25^

BOX 1 **Arrival and dispersal process in Wales**

**Table:** 

	Arriving in Wales as asylum seekers	Arriving in Wales with refugee status through resettlement programmes
Settlement	Initial temporary accommodation provided by National Asylum Support Service accommodation in one of six dispersal areas or housed in hotels. Often relocated to different locations several times. Will need to report to authorities (Home Office or Police) on a regular basis.	Settled anywhere in Wales through various national resettlement programmes. This could be in temporary locations such as hotels until private rented or local authority housing can be arranged.
NHS access (specialist/generalist)	In dispersal areas, local nurse‐led or general practitioner (GP)‐led specialist services conduct additional checks; ensure that asylum seekers are registered with a primary care provider; and signpost to other healthcare services such as dentists, opticians, maternity and child health services.	From time of arrival, access NHS services as the general population but do receive some short‐term support from voluntary and third sector organisations through the Vulnerable Persons Resettlement Scheme.
Health assessment	Offered initial health assessment, including tuberculosis screening and routine vaccination checks, by specialist asylum seeker nurses or specialist GP. After asylum claim is approved, specialist support ends. Access NHS as the general population.	Common expectation that refugees will have received a pre‐entry health assessment, usually at a refugee camp, as part of refugee eligibility, but this can vary, with often very little information provided.

This study was conducted across four Home Office asylum dispersal areas in Wales during the period from 1 April to 31 July 2018. At the time of the interview, there were 2869 asylum seekers receiving support across Wales.^26^

## 4 RECRUITMENT AND SAMPLING

We recruited and interviewed healthcare providers, support workers and practitioners from across primary, secondary, community and specialist NHS support settings over a 6‐week period. We purposively sampled through healthcare and third sector organisations known to the project team. We found chain‐referral (also known as snowball) sampling^27^ to be productive in securing a high response. In total, we had a list of 46 potential respondents, of whom eight declined to participate citing time constraints and six were unreachable. Eighteen respondents were from West Wales, five from South Wales, one from North Wales and eight from East Wales. Participants were located in four urban Home Office dispersal areas, one semiurban and two rural local authority areas. These areas comprised 56% of the Welsh population.^28^ With the aim of preserving anonymity and privacy of study participants, full names of these areas are not disclosed, nor did we collect data on participants' age, gender or work residence. Our chain‐referral approach to sampling is detailed in Box 2.

BOX 2 **Chain‐referral approach to sampling**

**Table:** 

Third sector partners and health providers provided contact details for (i) health assessment specialist service for newly arrived individuals and (ii) asylum seeker nurses and general practitioner (GP) services. These individuals suggested other services used by people seeking sanctuary such as maternity and dental services.
HEAR^21^ Research Management Group members including public contributors signposted to other providers including a pharmacist, health visitor, school nurse and specialist GP. Study interpreter suggested contacting a consultant in integrated sexual health.
Asylum seeker health services provided a list of GP practices, dentists and others used by people seeking sanctuary. We made visits, telephone calls and sent emails to several GP surgeries, dentists including pharmacies and opticians from this list.

## 5 DATA COLLECTION

We conducted qualitative telephone interviews using semistructured questions (see Supporting Information S1: File 1). The interview guide was developed by members of the Research Management Group (RMG) to explore issues highlighted in our literature review,^18^ on barriers and enablers to healthcare access by people seeking sanctuary. This included questions about the scope of healthcare provision, healthcare providers experiences and encounters and training and guidance. We audio‐recorded and transcribed these telephone interviews with verbal consent. Our research was conducted before the COVID‐19 pandemic, and during that time, workplace access to TEAMS or ZOOM was limited. Participation was voluntary, with no financial incentives offered to respondents. Interviews lasted between 30 and 50 min. Ethical approval was obtained from Swansea University Medical School Ethics Committee (reference number 2018‐0006).

## 6 ANALYSIS

We used framework analysis^29^, ^30^ informed by our study aims and interview guide to explore the perspectives of health providers and support workers on barriers to providing care for people seeking sanctuary. We then systematically explored the coded data for barriers and enablers to care using the four levels of change conceptual model to identify possible mechanisms for change. Two experienced qualitative researchers (A. K. and B. A. E.) independently read all transcripts and discussed initial themes. The main author further reviewed the data and organised them into a framework matrix using NVivo qualitative data analysis Software Version 12. The software allowed us to compare codes and themes and transcripts identifying consistent and inconsistent views among respondents to enhance inter‐rater reliability.^31^ Any differences in interpretation were resolved by A. K. and B. A. E. through rereading the original transcripts and field notes until they reached agreement on the results. The main author shared draft themes and write‐ups with members of the RMG for their input. Additional insights from the wider team of researchers, healthcare providers, Third sector organisations and public members from the sanctuary‐seeking community contributed to our understanding of how care is provided; they informed system wide and organisational workarounds to improve access to care.

## 7 RESULTS

Throughout the paper, we use the phrase ‘people seeking sanctuary’ to encompass all asylum seekers, people refused asylum and refugees. We recognise that certain issues impact all people seeking sanctuary, regardless of their specific position in the asylum or refugee ‘journey’. By using this inclusive term, we aim to shift the focus of discussions surrounding asylum and refugees back to individuals and communities directly affected by these challenges. When we encounter variations based on immigration status, we provide results using the relevant terms that describe their legal immigration status.

**Figure 2:**
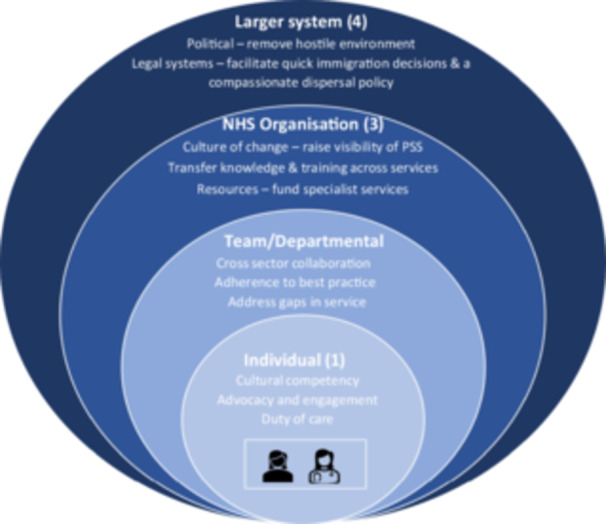
Adapted from Ferlie and Shortell,^23^ Four Levels of Change for Improving Quality.

We conducted 32 qualitative telephone interviews across Wales (see Table 1). To maintain anonymity, we identify quotations by groups^31^ (see Table 2). Figure 2 illustrates the Four Levels of Change for Improving Quality model at the individual, team/departmental, organisational and systems level. We applied this model to care processes identified through our study to ensure that comprehensive and coordinated responses could be provided to people seeking sanctuary. We report our findings according to four themes: (a) Scope of role affecting caregiving experience (Levels 2 and 3); (b) Level of awareness among health professionals of the care needs of people seeking sanctuary (Levels 1–3); (c) matching care needs and expectations to resources (Levels 1–3); and (d) legal status and access to services (Level 4).

**Table 1:** Respondent characteristics.

Categories	Group 1	Group 2	Group 3	Group 4
Location	Practitioners working in dedicated asylum seeker and refugee services	Practitioners in services with dedicated slots for asylum seekers and refugees	Practitioners in generic services accessed by asylum seekers and refugees living in the catchment area	Practitioners in generic services delivered in areas with low rates of asylum seekers or refugees
Roles	Asylum seeker nurse (*n* = 3)	Consultant/community dentist (*n* = 2)	Paramedic	Paramedic
	Refugee support worker (third sector)	Trainee dentist	Hospital overseas patient officer	Psychiatrist
	Community support worker (third sector)	Dental practice manager	GP	Palliative care nurse
	Specialist GP	Dental practice receptionist	Consultant in integrated sexual health	
	Health support worker (GP based)	Clinical director (dental services)	Trainee GP	
	Specialist health visitor	GP practice manager	GP receptionist	
	Specialist midwife	GP receptionist	Respiratory nurse	
			Community pharmacist	
			Opticians manager	
			School nurse	
			Health visitor (flying start)	
			Psychiatrist	
Total (*n* = 32)	9	8	12	3

Abbreviation: GP, general practitioner.

**Table 2:** Quotations from findings.

	*Scope of role affecting caregiving experience* (*Levels* 2, 3)
Specialist and support workers with access to tailored support services	23 (Grp 1 Lead Asylum nurse): they'll [people seeking sanctuary] often go from organisation to organisation trying to seek help. [My role] is just trying to coordinate that, to make sure that they are getting the right help from the right people.
	26 (Grp 3, specialist GP): need to provide a bespoke service. This is an equity issue not an equality issue, and if the needs are greater then the resources need to be greater. You'd need to introduce 30 minute appointments, appointments that can be missed without a problem and rebooked that same day, you'd need Big Word interpretation, to arrange same day investigations (x‐rays, bloods). All the letter writing and phone calls that we have to do with various Third Sector organisations. You'd need GPs to all be trained and understand how the Home Office works to be able to be flexible to write letters free of charge. To respond to destitution when it happens, when someone comes in and starts crying in front of you because they're now homeless.
	22 (Grp 3, Respiratory nurse): We work along with the Health Access team once every few months to do a clinic for TB screening.
	30 (Grp 2, Clinical Lead—Dentist): X (Asylum seeking nurse) would be referring people and telling us the background of the person in order for us to make a much smoother‐running service. She needs to be there, she's that person that leads in healthcare for us.
	10 (Grp 3, GP): Have specialist services set up in the same clinics. Support workers would be there, rather than sort of, erm, try and just dump them on general practice, who are trying to cope with a busy workload and you know, it's so difficult … Extend the time people can access specialist support services for maybe a year or so, they would have more time for them.
Access to healthcare provision	13 (Grp 1, Community support worker): If you haven't got photo ID it's not impossible but it's more difficult [for asylum seekers] to access dental care. GPs don't seem to mind so much. I think they accept there's a person stood in front of them with their name on a bit of paper. Dentists seem to want to see the photo ID. In fact, we've got one lady who famously—, by the time we actually pursued the Home Office enough for them to say they were going to issue it (ID Card), she had already got refugee status!
	4 (Grp 2, Opticians manager): I've got a few friends who are refugees myself. I know some of the procedures like, I ask them [people] if they've got an HC2 form.
	12 (Grp 3, GP Deputy Practice manager): We have clinical meetings every Monday, where the doctors talk about certain cases, often complex cases and, you know, very, very rarely do asylum seekers get mentioned at those meetings and just perhaps they stand out a bit by their absence, which is a shame.
	25 (Community Dentist): if a patient requires a very difficult orthodontic treatment that I feel it is not within my expertise there is difficulty with the asylum seekers how to guide them then to see a specialist.
	11 (Grp 3, GP Receptionist): if like there was one form in all Doctor's surgeries that was the same and there was a procedure that went with that form with better guidelines maybe, on how to register people. This would stop certain prejudices (by staff) and just them being awkward.
	27 (Grp 1, Refugee support worker): Because we're supporting, they [Health Board] expect that's something that we're going to pick up, rather than them [Health Board] having to find a way of helping the refugees.

	*Level of awareness among health professionals of the care needs of people seeking sanctuary* (*Levels* 1, 2, 3)
Awareness and understanding of need	28 (Grp 1, NHS support worker): They are very vulnerable patients and most of the times they just want to talk about how they feel or what they've been through. I think we shouldn't really focus just on health, but we should really focus on them as a person. I know my role is specifically just health but sometimes I do tend to ask, is everything okay? Is everything going smooth? That's how I end up referring them to other organisations.
	27 (Grp 1, Refugee support worker): I still feel they're having a lot of examinations, probably more so than a British person. Some of the doctors, yeah, we'll do CT scans, we'll do MRI scans, we'll do everything, but it's not a physical problem. It's a mental problem.
	12 (Grp 3, GP Deputy Practice manager): Once they are here, we have to make sure they get the services they need. Ultimately if they are not able to access primary care, then the burden will fall on secondary care and people are going to end up presenting to A&E very unwell.
	15 (Grp 3, Psychiatrist): basically, the screening should be done at the very basic level like, you know, when these patients come into their services they should have, you know, an assessment with the psychologist before the rest of the things can go on.
	4 (Grp 3, Opticians manager): asylum seekers, unfortunately do have a lot of eye conditions. Glaucoma's quite a big one. We went to the Sanctuary to do a talk. They don't know that children can have access … Like we can test a child's eyes and they don't have to speak English
	13 (Grp 1, Third sector support worker): I think they need specialist mental health support, because their needs are unique and very different from most of British society. Most of us haven't experienced war, most of us haven't experienced the trauma of slavery in Libya, most of us haven't had three days in a boat across the Med and seeing people drown. One guy who was here today, said, ‘Every night I dream that the Home Office refuse my case, send me back to Iran and that they hang me and my wife’. You know, and the only route we've got at the moment is to send him to a GP who will give him some tablets.
	10 (Grp 1, specialist GP): patients of mainstream surgeries can get access to other social prescribing support which allows them to cope with issues that asylum seekers share such as social isolation and unemployment. We can't access the Post Traumatic Stress Disorder clinic in secondary care because they're overwhelmed and they wouldn't be able to manage the workload if we were to refer everyone there. Plus the waits are 18 months, so it wouldn't be a necessarily appropriate, and we'd still need to manage them in the meantime. We can't access the National Exercise Referral Scheme, NERS. Because the providers can't accommodate the asylum patient due to the fact that there is a top up payment required at each visit. And they don't have translation facilities and various other things available, so there's not an outlet in terms of managing mental health concerns.
Communication	10 (Grp 3, GP): You feel very detached from the patient and from the interpreter when you're using a telephone.
	18 (Grp 3, Paramedic): some patients definitely end up in hospital inappropriately because of difficulties in communication, either establishing exactly how severe their complaint is or just the timeframe of getting translators and things involved and trying to find an alternative pathway especially for patients who haven't registered with GPs. So, though they don't always get the most appropriate care, they definitely still get the same good standard of care.
	12 (Grp 2, GP Deputy Practice manager): The asylum seeking nurse would send through to us (GP Practice) a little report about the person, but that was just kind of getting scanned and just put on the patient's notes and just kind of left there until their appointment. I wanted to make sure that all the information contained in those reports was actually being seen by the clinician where that was appropriate and that all the information was being coded and recorded in the notes properly.
	1 (Grp 1, Lead Asylum nurse): I've spoken to Managers in Primary Care, in Health Board they've thought that Pharmacists do use Language Line, so there's a real disparity there between—, on the ground what happens and what and what the Health Board think that they have
	31 (Grp 1, Specialist Asylum Midwife): I don't want her husband translating all the time. We need to‐‐, I need to use an interpreter so I know she's getting the correct information. I've never had an easy time getting hold of a language Line service. It normally takes at least an hour.
	15 (Grp 3, Psychiatrist): family members can be good interpreters. And, to be honest, I find them better than normal interpreters because they can give you a lot more information about the patient and how he's kind of acting at home as well.
	16 (Grp 3, NHS Overseas Officer): Yeah, well we—, the hospital does use Interpreters, but they do, obviously, cost money.
	27 (Grp 1, Refugee support worker): Most of the GP surgeries have never even heard of Language Line.
	14 (Grp 4, Palliative care nurse and volunteer): working in health care there is that, sort of, feeling that everything is rushed, and as soon as there's a language barrier it obviously makes the rushing feeling quite, sort of, frustrating.
Training	31 (Grp 2 Specialist Asylum Midwife): a lot of health professionals may never ever come across an asylum seeker in their daily work and it is such a specialised area, personally, as long as they have an overview, that's important but to go into the specifics around asylum, it's impossible, because it's just far too complex. I'm still learning everyday.
	5 (Grp 3, Consultant dentist): might ask people to do some role playing and that kind of thing.
	17 (Grp 3, Psychiatrist): offer standard training as part of the rotations with asylum seekers or with a refugee status that would be very interesting and very helpful.
	25 (Grp 2, Community Dentist): better understanding of where they've come from and their situation. The difficulties of their lives here or back in their country so we can understand their expectations for their dental treatment.
	1 (Grp 1, Lead Asylum nurse): Universities sometimes ask me to come in to speak to their students, so I'll go and just give an overview of, you know, the asylum‐seeking system and the impact on health and some of the health issues.
	11 (Grp 3, GP Receptionist): diversity training should be compulsory, especially in areas where you work with different people.

	*Matching care needs and expectations to resources* (*Levels* 1, 2, 3)
Resources and workarounds	2 (Grp 1 Lead Asylum nurse): Wales Audit Office actually use our services as best practice in the way we do communicate, in terms of giving our clients erm, letters with the picture of the clinic on the front, with a map. Our did not attend rate, the number of people that stopped attending fell dramatically.
	28 (Grp 1, NHS support worker): GPs, they felt under pressure … and the most voted support sought was for asylum seekers and refugees accessing support at the GP, they saw it as a need.
	28 (Grp 1, NHS Support Worker): there is a leaflet, a really handy leaflet actually, which basically is a checklist of everything that I provide as a health support worker for asylum seekers and refugees.
	18 (Grp 1, Dental Practice manager): unofficially, up in X area there was a dentist who had been arranging for the care of some asylum seekers through the Health Access team [specialist asylum seeker support service]. The Health Board wanted to formalise the care for asylum seekers. Under the contract that we are now, we can offer care using an interpreter, but I can imagine in a UDA (Unit of Dental Activity) practice yeah, people would probably try and avoid taking on patients with the language barrier.
	19 (Grp 3, Paramedic): In Birmingham we had a book and on the first page, there'd be, say, 20 languages and the patient would select their language and you'd go to that page, and there'd be a load of medical questions which I would point to in their language.
	4 (Grp 3, Opticians manager): Because of the language barrier, we try to make it as simple as possible and use keywords and use pictures instead of letters and ask them if that picture is bigger, smaller … We've also got a machine that tells and calculates the person's prescription as well.
	5 (Grp 3, Consultant dentist): It's difficult to get an NHS dentist anyway so what happens after we see them is we try to give the patients all the tools that they will need to look after their dentistry so dental disease is preventable.
	13 (Grp 1, Community support worker): Basically, we paid a trainer to come and deliver training. He's put together a four part programme that's aimed at supporting people through understanding that they've experienced grief. Really trauma is a form of grief, it's a sense of loss.
	14 (Grp 4, Palliative care nurse and volunteer): The English Speakers of Other Languages (ESOL) team have done a lot of English lessons about, you know, sort of, expressing your health concerns and, sort of, mock conversations with emergency services.
	14 (Grp 4, Palliative care nurse and volunteer): I was using Google Translate. I think life would have been very much easier a lot sooner if someone told me ‘Well, why don't you use the SayHi app?’
	1 (Grp 1 Lead Asylum nurse): I mainly get my information on asthma or hepatitis from NHS England, honestly I don't use NHS Wales. There's not that much out there, available.
	12 (Grp 2, GP Deputy Practice manager): We offer online access to appointment, but I guess for asylum seekers, their kind of housing situation and their internet access might not be as good as it could be. We open at eight o'clock in the morning and we try and encourage people to come to the surgery in person and wait.

	*Immigration policy* (*Level* 4)
Dispersal policy	30 (Grp 2, Clinical Lead—Dentist): they're moved on much quicker than they used to be … it does mean that sometimes they might have moved on before we even manage to get to see them.
Work barrier	6 (Grp 3, Consultant in integrated sexual health): Why don't they allow them to work? ‘Cause I was in Turkey for example, there are around 3 million refugees, and they are working everywhere. In the news they report yesterday or the day before somebody who was working washing cars and the police came and he jumped somewhere, fallen and died’.
	26 (Grp 3, GP): social isolation that comes with being removed from your usual community and not being able to work and the hostile environment created by the Home Office around their housing stability and the uncertainty around their asylum, the time for their asylum case to be heard is a big deal. We see a lot of gastric upsets, a lot of physical manifestations of stress. Some people don't eat well or sufficiently so we see people who are underweight or who have gastric problems from becoming quite hungry, many asylum seekers struggle with sleep so don't always turn up on time to their appointment.
Funding	12 (Grp 2, GP Deputy Practice manager): we're getting into the realms of politics, aren't we, and economics and things, which I know is a complex area, there should be more funding for these people, to improve their access.
Information	14 (Grp 4, Palliative care nurse and volunteer): Before a refugee arrives you have, sort of, a really vague outline. You, sort of, know, you know, the sex, age, any, sort of, major health needs, but you don't really get any nitty gritty, so I don't know if that's something maybe to flag up, that.

Abbreviations: GP, general practitioner; NHS, National Health Service.

## 8 SCOPE OF ROLE AFFECTING CAREGIVING EXPERIENCE (LEVELS 2 AND 3)

Among primary care practitioners, there was a range of experiences depending on whether their role was dedicated to supporting people seeking sanctuary or whether they provided care because sanctuary seekers were resident in the catchment area that they served.

### 8.1 Specialist and support workers with access to tailored support services

Specialists and support workers funded through the Local Health Board emerged as key care providers due to their expertise and practical experience in caring for people seeking sanctuary. These dedicated professionals provided both clinical and support services, tailored and delivered in places and at times that best align with people's needs. Their role involves guiding people seeking sanctuary through the health system, ultimately benefiting both patients and providers. These services would often work together with other providers, striving to co‐ordinate care effectively.

A general practitioner (GP) who provided care directly to sanctuary seekers reported that the flexible arrangements within her specialist service allowed her to provide tailored care to match her patients' specific needs. She perceived that her role extends beyond clinical care. She seamlessly integrates advocacy for (e.g., inadequate housing) and counselling services facilitated by her employers alongside clinical duties. In her opinion, this service was very different from mainstream general practice, where resources may not always align with the diverse needs of those seeking sanctuary.

### 8.2 Access to healthcare provision

Primary and community healthcare (PCHP) providers faced the challenge of integrating care for people seeking sanctuary into their existing workload, often with no new funding or resources. Although PCHPs clinical skills matched the role that they provided, many felt that more time or additional capacity was required to deliver care to patients attending their clinics. PCHPs recognised that sanctuary seekers had complex needs, as well as language barriers, which meant that routine appointment slots were too short. Many providers admitted knowing little about how to access interpreters or using telephone interpretation services. PCHPs reported various cultural differences such as gender norms, where men were not permitted to treat female patients, or different parenting expectations and female genital mutilation. While these are not direct barriers to treatment, they create challenges in resource management due to the need for additional time and specialised staff.

Some respondents expressed a strong moral and humanitarian responsibility to help, even though they acknowledged their lack of training, awareness of needs and resource constraints. They actively engaged with sanctuary seekers in informal settings to educate and encourage service use (e.g., optician service) and acquired knowledge through experience.

Nonclinical staff, such as practice managers and receptionists, were usually the first to interact with sanctuary seekers and manage their entry into the healthcare system. Challenges reported included insufficient support to process the necessary paperwork; questioning temporary accommodation as proof of address; difficulty finding suitable appointment slots and managing missed appointments; and stigma and discrimination. These challenges were exacerbated by Home Office delays in providing the correct identification. One respondent emphasised the disconnect between policy and practice. Despite recognising that people seeking sanctuary needed extra time and resources, their voice often remained overlooked in routine healthcare planning and review procedures.

## 9 LEVEL OF AWARENESS AMONG HEALTH PROFESSIONALS OF THE CARE NEEDS OF PEOPLE SEEKING SANCTUARY (ALL FOUR LEVELS)

### 9.1 Awareness and understanding of need, communication and training

Health providers' prior experience of supporting sanctuary seekers or their own personal empathy and compassion played a crucial role in recognising the vulnerability of people seeking sanctuary. They were committed to minimising the gap between need and service provision. To achieve this, they implemented changes in service delivery (e.g., interpreters available for consultations) or actively advocated for systemic improvements (e.g., prevent relocation during pregnancy).

Most PCHPs were unaware of specific training or resources to address their knowledge gap. Services such as language line (telephone interpretation service funded by Local Health Boards), which could enhance access to improved care, were not commonly known to them. To address their knowledge gap, some PCHPs sought informal guidance and training, often relying on specialised asylum seeker and refugee staff or by participating in locally commissioned training offered by Health Boards.

Respondents suggested that medical training for health professionals should include diversity training and understanding of the migrant experience. They also recommended having visible champions within key areas of healthcare planning to advocate on behalf of people seeking sanctuary.

## 10 MATCHING CARE NEEDS AND EXPECTATIONS TO RESOURCES (LEVELS 1–4)

### 10.1 Resources and workarounds

Specialist providers, with their broad scope and access to various clinical and person‐focused services, were able to align service provision with clinical requirements. While mainstream services understood the importance of person‐centred care, they frequently faced resource and training limitations in customising services for individuals. In certain regions, the demand for enhanced care prompted Health Boards to commission alternative services, such as NHS health support workers assisting general practice or funding dedicated dental services for sanctuary seekers.

PCHPs and support workers spoke about the complexities of maintaining continuity of care. One particularly challenging area emerged in the management of tuberculosis (TB). Language barriers and the young age of patients often hindered attendance or compliance with medication that was necessary to receive full TB treatment. To address these challenges, staff used different strategies to help them manage patients' needs, sometimes alternating between kindness and sanctions to deliver necessary clinical care. For instance, staff would facilitate transport arrangements and offered to process travel expenses, enabling patients to keep future appointments. However, this effort diverted time away from seeing more patients. While some PCHPs supported patients in practical ways, they refused to sign letters to the Home Office in support of asylum applications. In dental care, sanctions were more frequent. Dentist receptionists would remove patients, including sanctuary seekers, from their NHS register if they arrived late for appointments or missed appointments. These nuanced approaches aimed to balance care provision with practical constraints and administrative considerations but could deter access to care for those unfamiliar with the NHS system.

Poor access to mental health services emerged as a key concern among respondents. It was suggested that the health assessment for people seeking sanctuary should include a mental health assessment. This would ensure access to timely trauma‐informed care. However, respondents reported that sanctuary seekers lacked routine access to primary care mental health services because services were operating at full capacity. Even when available, these services were often ill‐equipped to address the specific trauma‐related or language needs of sanctuary seekers. It was reported that preventative NHS‐provided services such as exercise referral schemes or social prescribing opportunities were rarely offered to this vulnerable population due to language barriers. Third‐sector organisations often played a vital role in bridging this gap by offering nonmedicalised services like art therapy and gardening clubs to help people manage stress, anxiety and trauma.

## 11 LEGAL STATUS AND ACCESS TO SERVICES (LEVEL 4)

### 11.1 Dispersal policy, work barrier, funding and information

The circumstances of sanctuary seekers presented the greatest challenge for healthcare practitioners. Asylum seekers were liable to be moved without warning by the Home Office, which disrupted continuity of care. This posed significant challenges for individuals undergoing investigations or a course of treatment (e.g., chronic illness, TB or pregnancy and birth). Sudden relocations could take people away from trusted health providers as well as others they were connected to by culture, religion or language. Respondents also suggested that the lack of adequate services, combined with wider socioeconomic determinants of health such as social isolation, perceived discrimination, unemployment and inadequate housing conditions, contributed to poor mental and physical health. Respondents from all services reported that health outcomes and equity for people seeking sanctuary would improve if dispersal decisions considered the cultural, community and health circumstances of asylum seekers. Addressing these challenges required a holistic approach—one that considers not only medical care but also the unique sociocultural circumstances of asylum seekers.

Frequently, healthcare PCHPs were unaware that their patient would be a sanctuary seeker until they attended the first appointment. This lack of advance notice left little time for preparation. As a result, interpreters were not readily available, and additional appointments were often necessary to provide a comprehensive response. Some staff made efforts to stay connected with local specialist services who specifically looked after people seeking sanctuary. However, the sheer volume of appointments alongside the rising number of sanctuary seekers across all parts of Wales is challenging for PCHPs.

## 12 DISCUSSION

### 12.1 Summary

Healthcare professionals in Wales have identified significant challenges in meeting the needs of people seeking sanctuary. They report a mismatch between their capacity to provide treatment and support and the actual needs of individuals seeking healthcare. This discrepancy poses a considerable obstacle in delivering effective care to this vulnerable population. Specialist services and dedicated support workers had the advantage of having sufficient time, expertise and networks to provide person‐centred care. They could effectively coordinate clinical and social support for individuals seeking sanctuary. Staff in mainstream primary and community services report lacking resources and information. Their ability to tailor their services to specific circumstances and needs of sanctuary seekers is hindered. In some areas, services have collaborated and pooled resources. This approach has allowed them to provide specialist clinical services and recruit additional support workers. The ability to adapt and remain responsive to the needs of people seeking sanctuary is clear. However, challenges persist due to the effects of the asylum process. Specifically, antenatal care, mental health and TB management are areas where Home Office dispersal policies have disrupted the continuity of care. These interruptions can compromise the provision of optimal healthcare for those seeking sanctuary. It highlights the importance of addressing systemic barriers to ensure equitable access and consistent support for this vulnerable group.

Understanding the perspectives of health service providers and support workers perspectives is vital when exploring ways to improve the delivery of healthcare.^32^ Support workers and voluntary groups, particularly those dedicated to supporting resettled families, made considerable efforts to enable better access to healthcare for those they were supporting, despite not having prior information about people's health needs. They were often in regular contact and arranged many of the healthcare appointments and attended to other social and practical needs. While this level of personalised care appeared to be very beneficial for sanctuary‐seeking families, it is resource‐intensive in terms of time and effort. Opinions diverged on the impact of these specialist roles. One respondent suggested that these specialist roles removed the obligation on commissioners to invest in integrating sanctuary‐seeking services into mainstream provision; however, others called for extended specialist services to relieve pressure on already burdened primary and secondary care services.^33^

Respondents in general recognised the significance of their role. They actively addressed the complex health needs of individuals who had been affected by trauma and faced challenges settling in a new country.^33^ Notably, this is the first study in Wales to collect the views of care providers nationally. Furthermore, it contributes to one of the largest UK studies reporting healthcare provision and experiences among asylum seekers and refugees. The findings highlight the challenges in meeting the needs of individuals and offer valuable insights for enhancing care. Healthcare providers who appreciate the diverse cultural backgrounds of people seeking sanctuary understand the significance of acquiring the skills, attitudes and knowledge needed to deliver care that respects cultural norms, beliefs and practices.

### 12.2 Strengths and limitations

We acknowledge that healthcare providers' views can be diverse, influenced by their personal backgrounds, professional experiences and the specific contexts in which they operate in. While our research was conducted in Wales, the findings are likely to be applicable to other regions in the United Kingdom that are also subject to Home Office regulation on immigration. We used initial purposive and then chain‐referral approaches to identify a diverse range of respondents from both mainstream and specialist community health services. Our sample included health providers and practitioners from urban, semiurban and rural parts of Wales, working in various roles. We also engaged with third sector and volunteer‐delivered services. Some individuals declined to participate due to time constraints or perceived lack of experience in caring for people seeking sanctuary. This limitation suggests that our data may not fully represent all viewpoints and experiences across providers. Those who agreed to participate may have been more supportive of people seeking sanctuary compared to the general population of healthcare providers. Despite these limitations, our responses appeared to be generally consistent with views expressed in published literature contributing to the evidence base.

### 12.3 Comparison with existing literature

People seeking sanctuary, especially those who have experienced trauma and displacement, face significant challenges when it comes to accessing effective healthcare.^33^, ^34^ To address this complex issue, a conceptual framework was adapted from the Four Levels of Change for Improving Quality model proposed by Ferlie and Shortell.^23^ This framework aims to drive systemic improvements and is a useful tool to address challenges in delivering services to sanctuary seekers across health systems. In our study, these challenges have been reported across all four levels: legislative and financial administrative barriers that hinder effective service provision; lack of interpretation and culturally appropriate services, which can impede communication and understanding; lack of reliable information on illness and health histories of sanctuary‐seeking patients make accurate diagnosis and treatment challenging; lack of knowledge about healthcare entitlements and available services can hinder access; and finally, lack of organisation and coordination between services can lead to inefficiencies and gaps in care.^10^

Our research findings highlight the type of provision and routes to accessing care vary across regions, leaving sanctuary seekers more vulnerable if they are not located in an area where they can receive help from dedicated sanctuary seeker services.^34^ The absence of clear signposting information limits sanctuary seekers' ability to seek appropriate healthcare.^34^, ^35^ Simultaneously, healthcare providers' lack of information about sanctuary seekers' entitlements also restricts individuals' access to care and support.^36^

Our study respondents highlighted the significant impact of the legal and policy framework on their ability to provide care. The risk and uncertainty of relocation disrupt continuity of care and stability. Financial hardship and isolation disproportionately affect individuals with ongoing care for chronic or specialist conditions or pregnancy.^37^ Health professionals often have to navigate around tensions arising from Home Office policies and resource limitations within mainstream NHS services. This can impact their ability to provide care.^38^ Despite these obstacles, many professionals sought to adapt to these situations and identify workarounds. Additionally, cultural values and stigma within sanctuary‐seeking communities sometimes deterred individuals from seeking care, particularly related to mental illness, which made individuals fearful of seeking care.^34^

According to respondents, the mainstream primary and secondary health systems were too stretched to effectively accommodate the needs of sanctuary seekers.^33^ This had repercussions on the clinician–patient relationships and the overall functioning of the healthcare system (information, dissemination, training and linking with other services). Poor access to primary healthcare can potentially necessitate additional interventions including unscheduled and emergency care.^11^, ^39^ These issues are known to contribute to health inequalities and increase inefficiencies within healthcare services.^33^

### 12.4 Implications for research and practice

Healthcare should be accessible to all regardless of language abilities or additional needs. The findings from this study demonstrate that change is possible at the individual, team and/or departmental and organisational level affecting both clinical practice and policy. We found that some services have proactively recognised barriers and have taken initiatives to prioritise patient‐centred care by accommodating people within existing services (e.g., flexible appointment systems).

Requiring people seeking sanctuary to pay for services or restricting access to care until they can converse in English is an inappropriate response. Monitoring access to care and ensuring high‐quality interpretation during healthcare consultations are important components in facilitating access.^33^, ^40^, ^41^ Fostering two‐way unhurried communication^42^ applies not only during GP consultations but also extends more widely to interactions with health gatekeepers such as receptionists and wider primary care team members including opticians and community pharmacists.

Healthcare providers and support workers called for additional diversity training to understand cultural nuances,^43^ education around the migrant journey, trauma‐informed care^44^ and understanding entitlements to care. It was suggested that this training include nonclinical staff based in mainstream services, for example, receptionists and practice managers, to reduce barriers to receiving optimal care.

Specialist health professionals, third sector and community volunteers were a source of expert support for mainstream services.^21^, ^45^ It was noted that specialist services played a key role in supporting sanctuary seekers as they navigate unfamiliar care systems. They facilitated attendance at appointments and offered accessible health information. Their efforts contribute to a more equitable and effective healthcare experience for sanctuary seekers and alleviate pressures on an already burdened healthcare service.^10^, ^21^

In response, mainstream health providers could actively collaborate with specialist services to signpost patients to appropriate care. Greater cross sector collaboration would improve care delivery, enhance sanctuary seekers' experience, set realistic expectations, build trust with healthcare professionals and support continuity of care.^21^, ^42^ Healthcare providers and practitioners can also draw on available resources such as the British Medical Association's Refugee and asylum seeker patient health toolkit^2^ and the ‘Sanctuary seeker new to Wales?’ information pack^46^ for support.

The findings reported here can help address systemic challenges faced by healthcare professionals. Enhancing capacity, fostering better communication and collaboration and prioritising equitable care can help sanctuary seekers gain familiarity with healthcare services and attain and maintain better long‐term health outcomes.

## AUTHOR CONTRIBUTIONS

**Ashrafunnesa Khanom**: Conceptualisation; methodology; investigation; funding acquisition; formal analysis; project administration; writing—original draft; writing—review and editing; data curation; **Bridie A. Evans**: Formal analysis; writing—original draft; writing—review and editing. **Wdad Alanazy**: Investigation; data curation; writing—review and editing. **Lauren Couzens**: Conceptualisation; writing—review and editing. **Lucy Fagan**: Investigation; writing—review and editing. **Rebecca Fogarty**: Data curation; investigation; methodology; writing—review and editing. **Ann John**: Investigation; writing—review and editing. **Talha Khan**: Data curation; investigation; writing—review and editing. **Mark Rhys Kingston**: Validation; writing—review and editing. **Samuel Moyo**: Data curation; investigation; writing—review and editing. **Alison Porter**: Data curation; formal analysis; writing—review and editing. **Gillian Richardson**: Writing—review and editing; conceptualisation. **Grace Rungua**: Data curation; investigation; writing—review and editing. **Victoria Williams**: Data curation; investigation; writing—review and editing. **Helen Snooks**: Writing—review and editing; resources.

## CONFLICT OF INTEREST STATEMENT

The authors declare no conflicts of interest.

## Supporting information

Supporting information.

## Data Availability

The data that support the findings of this study are available on request from the corresponding author. The data are not publicly available due to privacy or ethical restrictions.
