# Correction to “Navigating Challenges and Workarounds: A Qualitative Study of Healthcare and Support Workers' Perceptions on Providing Care to People Seeking Sanctuary”

**DOI:** 10.1111/hex.70427

**Published:** 2025-09-17

Khanom A, Evans BA, Alanazy W, et al. Navigating challenges and workarounds: a qualitative study of healthcare and support workers' perceptions on providing care to people seeking sanctuary. *Health Expect*. 2024; 27:e14061. https://doi.org/10.1111/hex.14061

In the published article, the following author affiliations were listed as per below:

1. Wdad Alanazy, PhD, Lecturer Midwifery, Public Health Wales, Cardiff, UK
2. Lauren Couzens, Masters Public Health Senior Project Manager, University College Cork, Cork, Ireland
3. Lucy Fagan, Masters in Public Health Speciality Registrar in Public Health, British Red Cross, London, UK
4. Rebecca Fogarty, BA, Senior Project Manager, University College Cork, Cork, Ireland
5. Talha Khan, Medical Doctor, Public Contributor, Swansea, UK
6. Gillian Richardson, MPH, Assistant Director, Policy and International Health, World Health Collaborating Centre on Investment for Health & Well‐being, Public Health Wales, University College Cork, Cork, Ireland
7. Samuel Moyo Public Member—Asylum Seeker
8. Grace Rungua Public Member—Asylum Seeker

The correct author affiliations should read as per below:

1. Wdad Alanazy, Assistant Professor, Midwifery, PhD
2. Lauren Couzens, Senior Project Manager, Masters Public Health
3. Lucy Fagan, Speciality Registrar in Public Health, Masters in Public Health
4. Rebecca Fogarty, Senior Project Manager, BA
5. Talha Khan, Medical student
6. Gillian Richardson, Assistant Director, Policy and International Health, World Health Collaborating Centre on Investment for Health & Well‐being, Masters in Public Health
7. Samuel Moyo, Public member
8. Grace Rungua, Public member

We apologize for this error.

