# Supplementary material for: Navigating challenges and workarounds: A qualitative study of healthcare and support workers' perceptions on providing care to people seeking sanctuary
Source: Health Expect. 2024 Apr 28;27(3):e14061. doi: 10.1111/hex.14061 (PMC11056205; doi:10.1111/hex.14061)
Supplement: Supplementary file 1 — Supporting information. [file HEX-27-e14061-s001.docx]

**Supplementary material**

**Health providers, practitioners, and support workers (primary and Third Sector)**

**Aim**

As part of the HEAR study, we will conduct telephone interviews with health providers, practitioners and support workers to explore their experience of providing care for people seeking sanctuary and how they perceive it affects the care of people.

**Objectives**

To explore views on:

1. The scope of health provision and changing roles
2. Process and experience of delivering care to people seeking sanctuary including problems and benefits of providing care
3. Training received and how prepared they felt in providing and addressing the care needs of people seeking sanctuary in primary and secondary care
4. Their perceptions on the patients’ experience of care
5. Health care encounter
   1. Communication
   2. Cultural understanding (medication, access to health)
   3. Continuity of care
   4. Time
   5. Trusting relationships
   6. Health and social conditions
6. Health care system
   1. Linking in with other services; organisation and integration
   2. Professional support
   3. Resources and capacity
   4. Training and guidance

**Topic guide**

1. Please describe your professional role.
2. How often do you provide care for people seeking sanctuary in your line of work?
3. How would you know if someone was an asylum seeker or refugee?
4. How and why did you get involved with working with people seeking sanctuary?
5. How did this work/role come about? Is there specific funding available for this work?
6. Do you share the work load with any other colleague(s)?
7. How long have you been working this way?
8. Thinking about a recent contact can you describe how you responded to a patient who was an AS or refugee in your current role.
9. Refer to a particular situation
10. Assessment of the patient
11. Health problems
12. Which services do you often liaise with or refer patients to?
    1. GP
    2. Secondary care
    3. Social care services
    4. People seeking sanctuary specialist services?
13. Are there any issues you would like to talk about in terms of working with other health professional or social care professionals?
14. How do you think the patients respond to the care you provide?
15. What is it that you do that works well for the patient?
16. How willing are they to agree to your diagnosis/prescription
17. How do you manage their expectations?
18. How long do you support patients?

6. Thinking more generally about the care you provide;

a. Do you feel that you are confident in your understanding of the patients’ particular needs and entitlements?

b. Do you feel you have had the necessary training and resources to care for people seeking sanctuary patients?

c. Was the training sufficient?

d. Was the training long enough/too long?

e. How prepared did you feel after completing the training?

1. How long was it after the training before you were caring for people seeking sanctuary?
2. Was there anything else you would like to see included/done differently in the training?
3. How does the care that you provide to people seeking sanctuary differ compared to the general population?
   - 1. Time
     2. Resources
4. How do you think a health professional’s role should develop in the future to provide appropriate care for this patient group?
5. Do other health professionals share your views?
6. How do their opinions influence you?

9. Are there any specific issues you would like to raise about caring for this patient group?

i) cultural barriers/understanding (care, medication)

ii) practical/access issues

ii) communication

iii) risk involved with caring for this patient group

iv) health or social care issues

1. Is there anything else you would like to add?
